# Adjunctive remifentanil infusion in deeply sedated and paralyzed ICU patients during fiberoptic bronchoscopy procedure: a prospective, randomized, controlled study

**DOI:** 10.1186/2110-5820-2-29

**Published:** 2012-07-16

**Authors:** Hervé Quintard, Isabelle Pavlakovic, Jean Mantz, Carole Ichai

**Affiliations:** 1 Intensive Care Unit, University Hospital, Nice, France; 2 Anesthesiology Department, Beaujon Hospital, AP-HP, Paris, France

**Keywords:** Pain, Intensive care, Bispectral index, Remifentanil

## Abstract

**Background:**

Even with an adequate pain assessment, critically ill patients under sedation experience pain during procedures in the intensive care unit (ICU). We evaluated the effects of adjunctive administration of Remifentanil, a short-acting drug, in deeply sedated patient on variation of Bispectral Index (BIS) during a fiberoptic bronchoscopy.

**Methods:**

A prospective, randomized, blinded, placebo-controlled study was conducted in 18-bed ICU. Patients needing a tracheal fibroscopy under deep sedation (midazolam (0.1 mg/kg per hour) fentanyl (4 μg/kg per hour)) and neuromuscular blocking (atracurium 0.5 mg/kg) were included in the study. A continuous monitoring of BIS, arterial pressure, and heart rate were realized before, during, and after the fiberoptic exam. An adjunctive continuous placebo or Remifentanil infusion was started just before the fiberoptic exam with a target effect-site concentration of 4 ng/ml using a Base Primea pump.

**Results:**

Mean arterial pressure and heart rates were comparable between the placebo and Remifentanil groups at all times of the procedure. We did not observe differences in the variation of BIS values between the two groups during procedure. We described no change in BIS values relative to the placebo group in this population.

**Conclusions:**

In deeply sedated and paralyzed patients, receiving analgesic support based on a scale score an additional administration of short-acting analgesic drug, such as Remifentanil, seems not to be necessary for acute pain control.

**Trial registration:**

NCT00162591.

## Background

Pain, as it relates to care procedures, such as tracheal suction, mobilization, or wound care, is common in critically ill patients [1]. As such, it may have a negative impact on patient comfort and can contribute to the development of posttraumatic stress disorder at intensive care discharge. The Behavioral Pain Scale (BPS), by evaluating facial expressions, upper limb movements, and compliance with mechanical ventilation, has been developed and validated to assess pain in mechanically ventilated, noncommunicating patients [2,3]. Units that have implemented such pain monitoring procedures in daily patient care have reported decreases in hospital stays and in the number of ventilation days [4]. Even in properly sedated patients, however, variations on BPS scores have been described during a short painful procedure [5]. This suggests the need to optimize procedural pain control. Unfortunately in some situations, such as administration of muscle relaxants, this clinical scale cannot be used. Data indicate that variations of the Bispectral Index (BIS), initially developed to monitor the depth of anesthesia in the operating room, may be of interest to reflect cortical arousal associated with a painful procedure in mechanically ventilated, sedated, paralyzed patients [6].

Remifentanil, a short-acting opioid, is a particularly interesting option for the treatment of procedural pain in critically ill patients, due to its rapid onset and duration of action and metabolism independent of hepatic and renal status [7-9]. However, few data are available about its use in short painful procedure in ICUs [10].

The purpose of this study was to evaluate the effects of an adjunctive administration of Remifentanil on BIS variations during a short, painful procedure, such as fiberoptic bronchoscopy, in deeply sedated, paralyzed, mechanically ventilated patients.

## Methods

**Figure 1:**
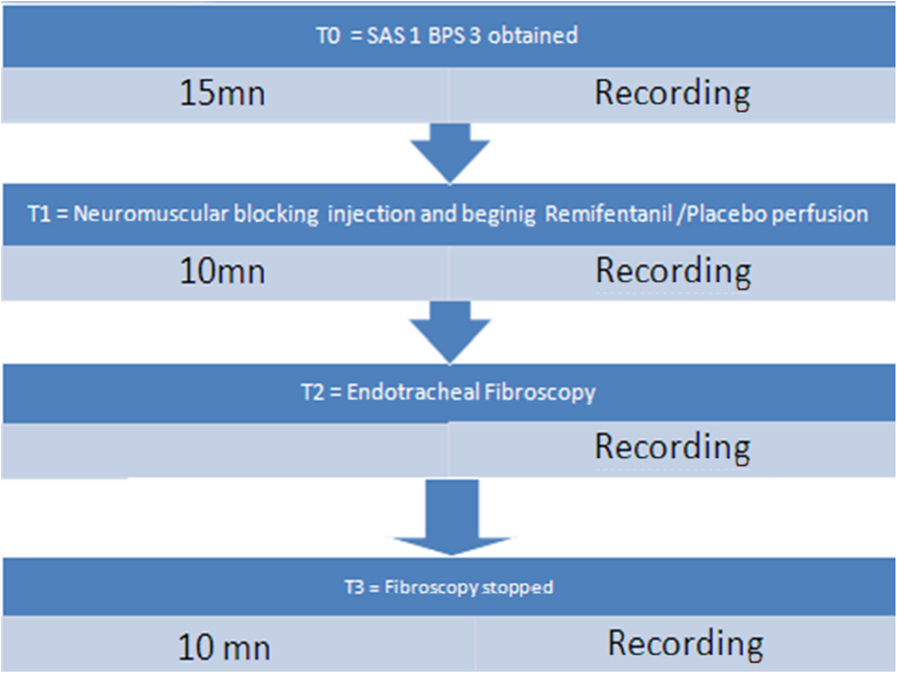
Study design.

This single-center (surgical and medical ICU recruitment), prospective, randomized, blinded, placebo-controlled study was approved by the Ethics Committee of the CHU of Nice, France (n° 06.027, Chairperson R. Collomp) on August 11, 2006, and written, informed consent was obtained from the families of the patients. Our study followed the CONSORT recommendations concerning the report of randomized trials. Inclusion criteria were: mechanically ventilated patient under sedation for Acute Respiratory Distress Syndrome (ARDS) needing an endotracheal fiberoptic bronchoscopy with alveolar lavage with use of muscle relaxants. Exclusion criteria were: evolving intracranial disease (brain injury, brain tumor, abscess, stroke, or hemorrhage). The sedation protocol was the same for all patients, and consisted of intravenous midazolam (0.1 mg/kg per hour) and fentanyl (1.5 μg/kg per hour). Drugs were administered continuously and their administration rates adapted step by step, ±0.03 mg/kg/h for midazolam and ±0.3 μg/kg/h for fentanyl, to the patient’s needs in accordance with a standard protocol using the Sedation Agitation Scale (SAS) (Appendix) [11] and the Behavior Pain Scale (BPS) [5] before neuromuscular blocking. This strategy was in accordance with the 2007 French Society of Critical Care guidelines [12]. The SAS and BPS targets before neuromuscular blocking were 1 and 3 (T0), respectively. BIS values were continuously recorded using BIS-XP (software version 3.12), developed by Aspect Medical System®, and routine hemodynamic monitoring was performed with the Philips® Intellivue monitor. When the SAS and BPS goals were reached, BIS was continuously recorded for 15 minutes (LB0-15). Patients were unrestricted randomized, in a double-blinded fashion, into two groups: placebo or Remifentanil group. Then, a neuromuscular blocking agent (atracurium 0.5 mg/kg) was injected in minutes to minimize nonspecific histamine release. Neuromuscular blocking agents were systematically used to optimize ventilation during procedure and for optimization of the fiberoptic bronchoscopy [13]. Train of Four (TOF) responses of the left and right orbicularis were recorded (C0-C10). At the same time (T1), either Remifentanil or the placebo was given at a target effect-site concentration of 4 ng/ml using a Base Primea pump (Fresenius-Vial®, Brezins, France). The concentration of 4 ng/ml was chosen in view of previous data showing that higher concentrations can be associated with hypotension and bradycardia [14-17]. Ten minutes later, the endotracheal fiberopticbronchoscopic procedure was started (T2) (FB 0-FB 20). BIS was recorded until 10 minutes after the end of the procedure (T3) (PF 0-PF 10) (Figure 1).

All statistical analysis of data was performed by using StatView software. Results were calculated for groups of 20 patients, following a previous study [6], to obtain a 50 % decrease in the BIS value after tracheal suction in treated patients with a power of 80 % and α of 0.05. Results were expressed as mean (±SD), and all data were tested for normal distribution by the Kolmogorov-Smirnoff test. Differences between the pre-procedure and maximum BIS values for a given fiberoptic bronchoscopy were compared between the two groups by Student’s *t* test. BIS, heart rate (HR), invasive mean arterial pressure (MAP), SpO_2_, and CO_2_ for each time and changes of these parameters during the procedure were analyzed using an one-way (or two-way) ANOVA for repeated measures analysis.

## Results and discussion

### Demographic data

Forty patients presenting criteria for ARDS defined by the American-European consensus conference [18] were included in this study between January 2008 and January 2009. One patient was excluded because of missing data. No adverse effects related to Remifentanil infusion occurred. There were no differences in age, weight, height, gender, length of sedation, or median dose of midazolam and fentanyl before procedure between the two groups (Table 1). All of the patients had SAS and BPS scores of 1 and 3, respectively, before the use of muscle relaxant. The overall agreement between BIS value and clinical sedation assessment made with SAS and BPS was evaluated. All the patients had deep level of sedation controlled by SAS and BPS before procedure but high BIS values (>60) were present in nine cases. BIS values decreased lower than 60 after the administration of atracurium agent for all patients.

**Table 1:** Demographic data (mean ± SD)

	**Placebo group**	**Remifentanil group**	***p***
Age (yr)	56 ± 18	51 ± 17	0.2
Apache score	17 ± 12	15 ± 9	0.5
Ratio male/female	18/2	17/3	0.1
Weight (kg)	72 ± 29	78 ± 14	0.2
Height (cm)	156 ± 54	175 ± 7	0.2
Length of sedation before fiberoptic act (days)	3 ± 2	4 ± 3	0.1
Median Midazolam dose before procedure (mg/h)	11 ± 3	13 ± 4	0.2
Median Fentanyl dose before procedure (μg/h)	150 ± 50	165 ± 45	0.2
Maximum BIS variation during procedure	15 ± 14	12 ± 10	0.5
Median total dose of Remifentanil during procedure(μg)	142 ± 95	155 ± 70	0.3

### Physiologic recording

**Figure 2:**
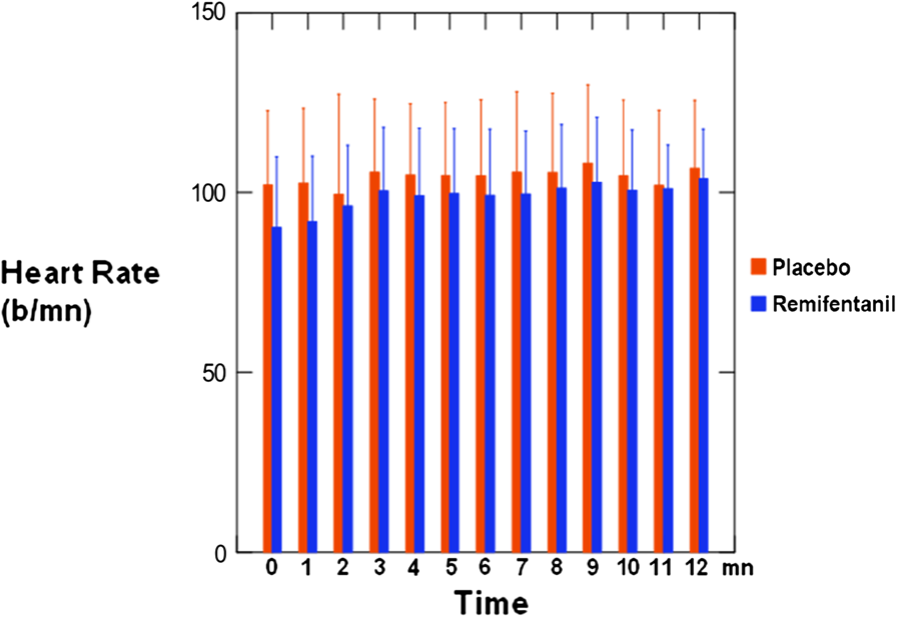
Mean heart rate (±SD) recorded each minute during procedure.

**Figure 3:**
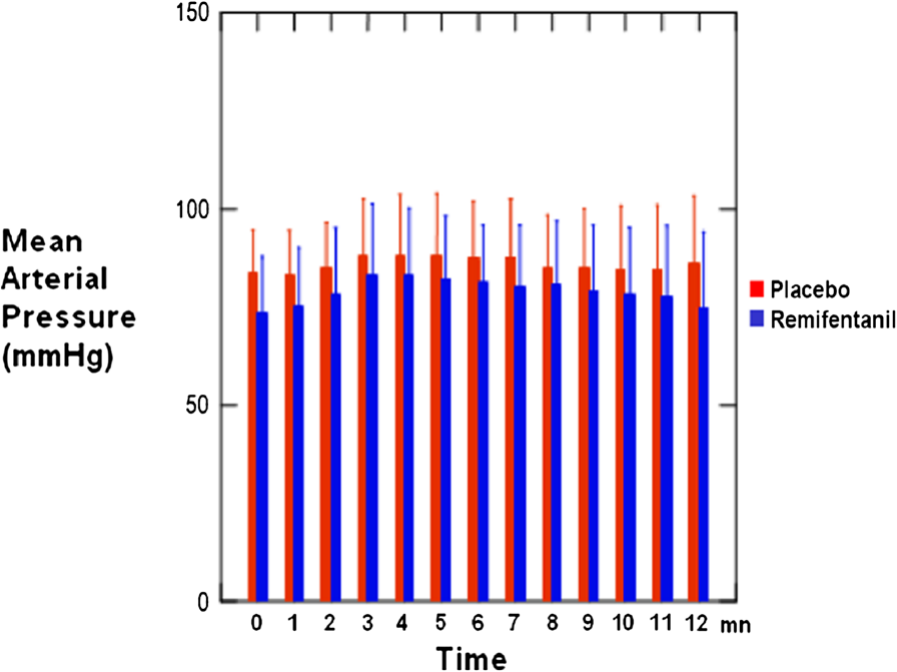
Mean arterial pressure (±SD) recorded each minute during procedure.

The median recording time during the procedure was 12 ± 4 min, with no difference between the groups. Heart rates and mean arterial pressure were comparable between the placebo and Remifentanil groups during fiberoptic bronchoscopy procedure (Figures 2 and 3). We observed no SpO_2_ and CO_2_ variations during procedure.

### BIS recording

**Figure 4:**
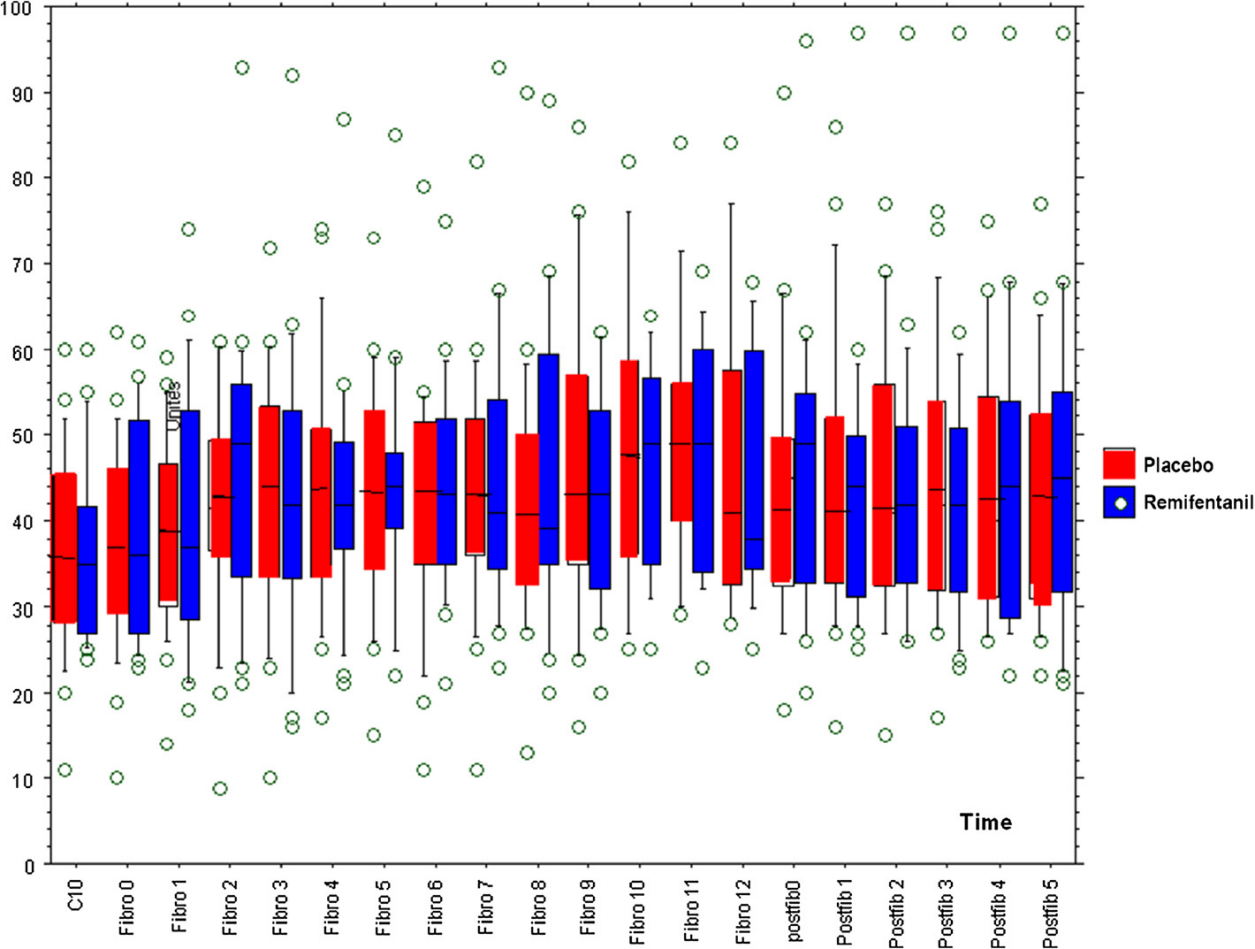
**Box plot of BIS variation during procedure with mean (box plot) with median, interquartile (25**^**th**^**–75**^**th**^**), minimum and maximum values and outliers.** C10 10 mn after the myorelaxant injection.

**Figure 5:**
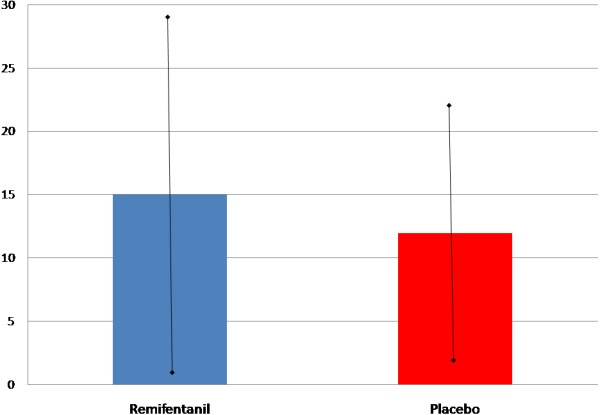
Maximum BIS variation during fiberoptic bronchoscopy (mean ± SD).

The mean BIS values before the fiberoptic bronchoscopy were not significantly different between the two groups: 36.2 ± 11.9 vs. 35.8 ± 10.9 (*p* > 0.05) (Table 2). Similarly, we observed no differences between the two groups in the variation of the BIS value during the procedure (Figure 4). Specifically, relative to the values determined before the procedure, neither BIS nor maximum BIS variation were different during the fiberoptic bronchoscopy, between the two groups (15 ± 14 and 12 ± 10; *p* > 0.05; Figure 5).

**Table 2:** BIS monitoring (mean ± SD)

	**Placebo**	**Remifentanil**	
**BIS value before fiberoptic bronchoscopy**	36 ± 12	36 ± 11	>0.05
**Maximum BIS value during procedure**	53 ± 8	54 ± 11	>0.05
**BIS value post fiberoptic bronchoscopy**	42 ± 6	46 ± 9	>0.05

## Discussion

We report in this study that adjunctive boluses of remifentanil are not necessary to prevent acute pain in deeply sedated patient.

Protocolized optimized sedation and pain control in the intensive care unit (ICU) can help to reduce duration of mechanical ventilation and length of stay in ICU and hospital by reducing drug consumption [4,19]. However, it could be not sufficient to provide an adequate analgesia during an acute painful care. Indeed, Payen et al. described a rising of BPS during procedure in patient under correct level of sedation [5]. Optimization of analgesic control, by adjunctive therapy for example, could be needed to impair the risk of posttraumatic stress. Remifentanil, because of its pharmacologic properties could be a very interesting approach. This short-acting drug could protect the patient during procedure without increasing the risk of oversedation and consequences. In this study, we did not observe differences in the variation of BIS values, as a surrogate for analgesia evaluation, between the Remifentanil and placebo groups in mechanically ventilated, deeply sedated patients during a fiberoptic bronchoscopy. Moreover, BIS value did not change over time in both groups. We also do not observe significant difference between placebo and Remifentanil group according to PAM and heart rate. Heart rate is always higher in the placebo group than in the remifentanil one, which could be explained by direct chronotropic negative effect of Remifentanil. The absence of BIS variation observed during the procedure could be explained by different ways. The very low values recorded at the beginning of the procedure can limit the impact of adjunctive therapy. Indeed, we choose to study a particular group of patients needing deep sedation for procedure. We may hypothesize that deep sedation conducted to low initial BIS value, leading to blunt significant variation in BIS value. Statistically, an expected decrease in BIS value of 50 %, used in anterior studies, is probably overestimated in this particular setting of deep sedated patients, and the sample size could be underevaluated. Tolerance to fentanyl developed before the procedure could limit the interpretation of results, but length of sedation before procedure was short and dose was controlled by pain scale. Furthermore, no signs of opioids tolerance were developed by patients. We can also hypothesize that the level of pain is not sufficient but previous study described fiberoptic examination as one of the most painful procedure [1]. Remifentanil doses could not be enough efficient, but the absence of BIS variation in the placebo group is not in accordance with this hypothesis.

In particular, critically ill conditions, such as patients with brain trauma or respiratory failure, or for care procedure (fiberoptic bronchoscopy, tracheotomy…), neuromuscular blocking agents administration is recommended making the use of sedation scale scores, such as the Behavioral Pain Scale (BPS) [5], impossible. Bispectral Index, developed initially to monitor depth of anesthesia in the operating room [13,20,21], could be an approach. In an experimental study of healthy volunteers, adjunctive opioids to volatile agents-based anesthesia increases clinical sedation but has no impact on BIS value. However, patients did not suffer from acute painful procedure [22]. In ICU, BIS use remains controversial because of the high level of variability of this parameter as described in our study (Figure 4), which led us to study the variation of this rather isolated value. Some authors have concluded that the most recent version of BIS—BIS XP—is useful in the ICU setting for assessing sedation [23], whereas others do not share this view [24]. The use of BIS monitoring to assess pain in our study could be criticized, but it was shown to be sensitive to nociceptive stimuli in critically ill, sedated patients [6,25]. Brocas et al. were able to blunt BIS variations by adding a bolus of a short-acting opioid to sedated patients before endotracheal suction. Other studies have described similar results with other opioids [17]. In accordance with these results, and with the proposition of French consensus on sedation in the ICU, we decided to use this device to evaluate the level of pain of our critically ill patients [12].

## Conclusions

Our study shows that in deeply sedated patients receiving analgesic support based on a scale score, additional administration of short-acting analgesic drug, such as Remifentanil, does not seems to be necessary for acute pain control assessed by BIS variation. These data should be studied further in a population of patients who need less sedation.

## Appendix

### A.1. Riker sedation-agitation scale (SAS)

#### *A.1.1. Score term descriptor*

7 Dangerous Agitation Pulling at ET tube, trying to remove catheters, climbing over bedrail, striking at staff, thrashing side-to-side

6 Very Agitated Requiring restraint and frequent verbal reminding of limits, biting ETT

5 Agitated Anxious or physically agitated, calms to verbal instructions

4 Calm and Cooperative Calm, easily arousable, follows commands

3 Sedated Difficult to arouse but awakens to verbal stimuli or gentle shaking, follows simple commands but drifts off again

2 Very Sedated Arouses to physical stimuli but does not communicate or follow commands, may move spontaneously

1 Unarousable Minimal or no response to noxious stimuli, does not communicate or follow commands

## Abbreviations

SAS: Sedation agitation scale; BPS: Behavioral pain scale; BIS: Bispectral index.

## Competing interests

The authors declare that they have no competing interests.

## Authors’ contributions

HQ conceived the study and participated in its design, coordination, and drafted the manuscript. IP participated in acquisition of data. JM participated in its design, coordination, and helped to draft the manuscript. CI has given final approval of the version to be published.
